# Three-Dimensional Printing of Living Mycelium-Based Composites: Material Compositions, Workflows, and Ways to Mitigate Contamination

**DOI:** 10.3390/biomimetics8020257

**Published:** 2023-06-14

**Authors:** Alale Mohseni, Fabricio Rocha Vieira, John A. Pecchia, Benay Gürsoy

**Affiliations:** 1 Department of Architecture, Penn State University, University Park, PA 16802, USA; 2 Department of Plant Pathology and Environmental Microbiology, Penn State University, University Park, PA 16802, USA; vieira.cogu@gmail.com (F.R.V.); jap281@psu.edu (J.A.P.)

**Keywords:** mycelium, 3D printing, mycelium-based composites, waste cardboard, waste paper

## Abstract

The construction industry makes a significant contribution to global CO_2_ emissions. Material extraction, processing, and demolition account for most of its environmental impact. As a response, there is an increasing interest in developing and implementing innovative biomaterials that support a circular economy, such as mycelium-based composites. The mycelium is the network of hyphae of fungi. Mycelium-based composites are renewable and biodegradable biomaterials obtained by ceasing mycelial growth on organic substrates, including agricultural waste. Cultivating mycelium-based composites within molds, however, is often wasteful, especially if molds are not reusable or recyclable. Shaping mycelium-based composites using 3D printing can minimize mold waste while allowing intricate forms to be fabricated. In this research, we explore the use of waste cardboard as a substrate for cultivating mycelium-based composites and the development of extrudable mixtures and workflows for 3D-printing mycelium-based components. In this paper, existing research on the use of mycelium-based material in recent 3D printing efforts was reviewed. This review is followed by the *MycoPrint* experiments that we conducted, and we focus on the main challenges that we faced (i.e., contamination) and the ways in which we addressed them. The results of this research demonstrate the feasibility of using waste cardboard as a substrate for cultivating mycelia and the potential for developing extrudable mixtures and workflows for 3D-printing mycelium-based components.

## 1. Introduction

The world is currently facing the threat of a major environmental disaster, and the construction industry plays an important role in the current conditions. The construction industry must undertake considerable change to achieve sustainable practices and a circular economy. The consumption of non-renewable building materials should be minimized [1]. According to the United Nations, the construction industry is responsible for 38% of global CO_2_ emissions due to energy consumption, and material extraction, processing, and discharge have the greatest environmental impacts in this industry [2]. There is an increasing need for sustainable building materials, and the focus is now shifting toward the development and implementation of novel biomaterials that support the concept of a circular economy over their life cycle [3]. Biomaterials are abundant on both local and global scales, have a low carbon footprint, and are biodegradable. Designers and architects, in collaboration with material scientists and biologists, have explored how to design and fabricate using biomaterials [4]. The mycelium is fungi’s hyphal system, and its inadequate growth forms mycelium-based composites [5]. These composites have the ability to transform bio-waste into high-end goods. When the adaptive growing nature of mycelial networks is combined with the potential to shape matter in three dimensions, complex living materials that meet particular engineering needs can be produced. Mycelium-based biomaterials need to be carefully managed and grown in a controlled environment to form functional structures. This requires transforming biomaterials’ shape or adding substances that enhance their characteristics. To develop the appropriate structures, their growth and development must be monitored and regulated as living organisms [6]. Mycelium-based composites provide good heat and sound insulation, are hydrophobic, have compression resistance, are 100% biodegradable, and have low density. Furthermore, the raw ingredients for such composites are low cost, locally available, renewable, and capable of capturing and storing CO_2_ [7]. Cultivating mycelium-based composites utilizing the molding technique is a common approach [8]. However, molds limit the geometric complexity and scale of mycelium-based composites. Furthermore, if molds are not reusable, the process is often wasteful.

Three-dimensional printing methods, as an alternative to molding techniques, can be used to form mycelium-based composites, allowing unique shapes to be manufactured without the need for unique molds [9]. In addition, this can minimize waste from the manufacturing process and make it possible to create complex shapes that can support the growth of mycelia that would otherwise be impossible to produce [10]. The integration of the livingness characteristic of mycelium-based biomaterials and the shaping potential of 3D printing technology opens up the possibility of developing living materials with unprecedented adaptive features [6]. This study focuses on developing 3D printing workflows and extrudable mixtures with mycelium-based composites that are cultivated on waste paper, waste cardboard, and waste newspaper, as well as addressing ways to mitigate a major challenge of the 3D printing process, contamination.

## 2. Background

Biomaterials have caught the attention of architects and designers, resulting in a considerable increase in the number of projects that have been carried out over the last decade [3], proposing long-term solutions to issues such as fossil fuel dependency, greenhouse-gas emissions, and solid waste production. Biomaterials are materials that include at least one component that has been formed biologically and are completely biodegradable [1].

### 2.1. Mycelium-Based Composites

**Figure 1:**
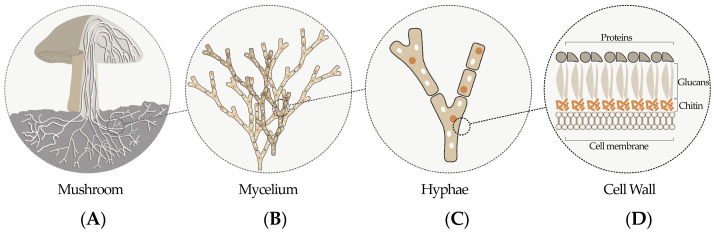
On various scales, a schematic representation of the mycelium is shown: (**A**) mushroom structure, (**B**) hyphae, (**C**) several cells of hyphae, and (**D**) single hyphal cell wall (adapted from [14]).

Mycelium-based composites are biomaterials that are formed when living fungi grow onto organic substrates, which also act as nutrition sources for the fungi [11]. The mycelium, a network of fungal hyphae, is composed of a network of “fine fibers” of 1–30 μm in diameter [8,12]. Chitin, glucans, and proteins are the main components of the hyphal wall, as illustrated in Figure 1. In particular, chitin is an intricate polysaccharide that consists of modified glucose chains and is primarily responsible for the material properties of mycelium-based composites [13]. A fungus takes nutrition from its environment using its mycelium. In order to break down the biological polymers of organic materials into simpler molecules, the hyphae first discharge enzymes into the organic substrates and subsequently absorb the resulting monomers. When the mycelial branches combine the organic substrates in this manner, the result is a lightweight and foam-like substance, called mycelium-based composite [5]. With the development of their mycelial network, fungi can integrate various types of organic waste into composite materials without requiring extra energy or creating additional waste [8]. Mycelial cells have evolved in nature to maneuver through and develop within the hollow spaces of porous structures. This metabolic activity gives them the strength to self-regenerate and repair the broken or empty spots in mycelium-based composites [6].

As living organisms, fungi react to environmental influences by rebuilding their hyphal network to optimize nutrient intake. They transform continuously, resulting in different properties across the mycelial structure. In the presence of appropriate substrates, the mycelial structure can self-replicate to generate seemingly limitless composites [15]. Mycelium-based composites are foam-like, light-weight materials formed by drying or heating the mycelial colony, which ultimately results in the hibernation or death of the fungal mycelium [5]. According to [6], the relationship between exploration and exportation implemented by different fungal species can explain the different growing patterns of mycelial networks. There are two different growing strategies based on the availability of nutrients, known as phalanx and guerilla. When a large, easily available nutrition source is available, the mycelium uses an exploitative growing strategy, also known as phalanx, by progressing slowly in a continuous line, which results in a thick hyphal mat with many branches. On the contrary, when the nutrition source is limited, the mycelium changes the growing pattern to an exploratory mode, known as guerilla, resulting in long, branchless hyphae [6].

Although frequently referred to as mycelium-based composites, these biomaterials are also called myco-materials, fungi-based materials, mycelium materials, mycelium composites, and mycelium-bonded composites in the literature. In this paper, we adopt the term mycelium-based composites.

Multiple factors affect the mycelial growth and the material properties of the resulting composites. These include the species of fungi utilized for inoculation, the substrates and additives used, the growing conditions, the cultivation period, and the method of processing and formation [16]. By altering these determining factors, researchers attempt to enhance the mechanical and durability characteristics of mycelium-based composites [17].

Recently, there has been a growing interest in using mycelium-based materials in architecture. Architects working on digital and computational design analyze and decode molecular structures, such as the hyphal network of mycelia, to learn about the geometric, structural, and behavioral motions [3]. Mycelium-based composites can be formed using a variety of methods and are used to construct a wide range of architectural elements [4].

### 2.2. Three-Dimensional Printing of Mycelium-Based Composites

One of the distinctive qualities of mycelium-based composites is the ability of mycelia to grow in molds, which enables designers to directly cultivate composites in the final, desired form. This has been the preferred method to construct architectural prototypes so far [18]. The molding process proceeds with the following stages: the substrate and fungal inoculum (spawn) are mixed, placed in a sterilized mold, and allowed to develop until mycelial growth is ceased using heating or drying [11]. While this method is rather straightforward, it is well suited for industrial-scale production. However, molding techniques limit the product’s customization and geometrical complexity [19]. Furthermore, molds can produce waste, especially if they are not reused or recycled. Recently, there have been efforts to use additive manufacturing techniques to form mycelium-based composites, which are reviewed in detail and comparatively in Section 2.3.

The 3D printing of mycelium-based materials can facilitate the fabrication of more intricate forms without the need for unique molds and provides new applications for mycelium-based composites [7]. According to Soh et al. [11], extrusion is one of the least energy-intensive manufacturing techniques, making it a promising option for the low-cost and low-energy manufacture of mycelium-based products.

In order to 3D-print mycelium-based composites, it is important to first develop an extrudable paste that has form stabilization qualities and is workable and buildable. To improve workability and buildability, additives, such as fibers and binders, are commonly used. Altering the water content and using modifiers to change the mixture’s viscosity is also an option [11].

### 2.3. Three-Dimensional Printing Workflow for Mycelium-Based Composites

**Figure 2:**
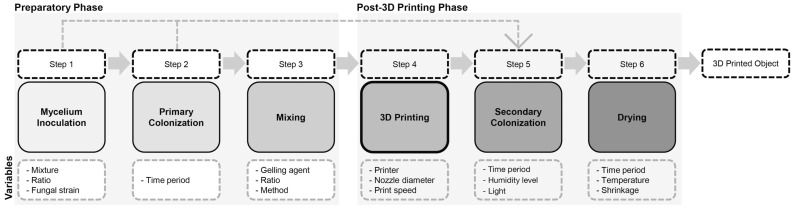
Six steps of the 3D printing of mycelium-based bio-composite material (adapted from [7]).

Mycelium-based composites for architectural use can be grown on a variety of substances and under a variety of conditions. There are three main stages of mycelium-based composite cultivation: inoculation, growth, and ceasing. Based on studies in which extrusion techniques have been already developed for mycelium-based composites, the steps for 3D printing mycelium-based composites can be divided into two primary stages, before and after 3D printing [7,11]. The entire 3D printing workflow can be divided into six steps: mycelium inoculation, primary colonization, mixing, 3D printing, secondary colonization, and drying [7,20], as shown in Figure 2.

#### 2.3.1. Step 1: Mycelium Inoculation of Agricultural Feedstock

Inoculation is the first step in the cultivation of mycelium-based materials. It starts with the preparation of the substrate mixture. Mycelium-based composites have two main parts: a filler, that is, a bio-substrate, and a binding agent, that is, a mycelium. Mycelium-based composites can be compared to concrete that has aggregates and cement, but the main difference is that in mycelium-based composites, the substrate works as a source of nutrients for the fungi for the next stages of development [21]. Mycelium-based composites may be cultivated on a wide range of organic matter. Based on the literature, the most common substrates for developing mycelium-based composites are straw, woodchip, and hemp [17]. On the other hand, cardboard, paper, and newspaper have a high content of plant materials, such as cellulose, hemicellulose, and lignin, which can easily be degraded and used as carbon sources by primary decomposer fungi [22].

To eliminate any possible microbial contaminators, the mixture must be sterilized [23]. Sterilization should be performed before the substrate is inoculated with the fungal mycelium. There are several methods, such as cold sterilization with chemicals, scalding, using peroxide, cold fermentation, and sterilizing in bags in an autoclave. The most common approach is using autoclavable bags filled with the prepared mixture and sterilizing in an autoclave for 30–45 min at 121 °C or at lower temperatures, such as 90 °C, for a longer period [23]. The fungal spawn is added after the mixture has been sterilized and cooled to eliminate any microbial competitors. The fungal species is a determining factor of mycelial behavior during growth as a binding agent. It is critical to choose the appropriate fungal species to achieve the desired material quality in the end [24].

#### 2.3.2. Step 2: Primary Colonization

Fungi grow in the form of colonies on primary substrates or host materials, which is why this step is called primary colonization [7]. Colonization is the second step of the cultivation process, during which the mycelium goes through different development phases. In most cases, this initial step of mycelial formation occurs in the sterilization bags. The colonization procedure takes approximately between 5 and 15 days, depending on the substrate type, fungal species, and growing conditions [17]. During the first stage of development, little branches of hyphae form in the bags, mostly on the surface of the mixture [5]. Once colonization is visible, the mixture is ready to be taken out of the bags for the next steps. If there is enough and equal growth in the mixture, it is possible to proceed to the next steps of the cultivation, which include molding the mixture into the desired forms or adding some admixtures to make it extrudable for the 3D printing method. This step results in a dense mixture [7], the characteristics of which can be altered by changing the environmental factors, such as temperature, humidity, and light, throughout the cultivation process [5].

#### 2.3.3. Step 3: Mixing

Before extruding mycelium-based composites, it is necessary to develop a paste or mixture that is extrudable and has certain characteristics, such as flowability, rheology, and buildability [11]. Flowability is one of the key measurements for ensuring that the paste is successfully delivered from the container to the nozzle during 3D printing. Rheology is linked to material flow and deformation, which, in the case of printable materials, is the examination of printability, buildability, and segregation resistance. Buildability, on the other hand, is the capability of a material to hold its printed shape and the durability of an extruded wet material against distortion under its self-load [25].

The primary colonized material is initially too thick for passing through the print nozzle. To convert the thick colonizing-mycelium-based composite into an extrudable mixture that is flowable and buildable, more water and a gelling agent must be added [7]. The ingredients can be mixed using a mixer to enhance their printing quality and integrity.

#### 2.3.4. Step 4: Three-Dimensional Printing

The determining factors of 3D-printed mycelium-based composites can be divided into two main categories: (1) factors associated with the material itself, including material particle size, viscosity, and ingredient concentration; (2) factors associated with the printing environment and printing equipment, such as extrusion pressure, extrusion speed, nozzle diameter, toolpath geometry, and the 3D printer [26].

#### 2.3.5. Step 5: Secondary Colonization

The living mycelium existing in the mixture must expand its growth to solidify the composite after 3D printing [11]. According to Bhardwaj et al. [7], secondary colonization refers to this second stage of mycelial growth, where the fungi begin to proliferate again. This period varies based on the fungal species used for the first colonization step. The 3D-printed objects must be maintained in sterilized containers away from direct sunlight to support further fungal development.

#### 2.3.6. Step 6: Drying/Heating

To stop the growth process of mycelium-based composites, it is necessary to either dry or heat the composites [27]. This step takes place after secondary colonization. Drying hibernates the fungus; if the material is exposed to the desirable humidity conditions again, it may restart growing. Adequate heating, on the other hand, kills the fungus and terminates further colonization by the mycelium.

### 2.4. Review of Previous Studies on 3D Printing of Mycelium-Based Composites

Previous experiments on the 3D printing of mycelium-based materials can be divided into two groups, large-scale and small-scale prototypes. There are currently two large-scale projects to date built by 3D-printing mycelium-based composites, where both of them are column-like vertical structures: Pulp Faction and the column by Blast Studio. Both projects were developed using a reaction–diffusion algorithm for form finding [28]. After the completion of the first phase of growth in bags, the manufacturing process of both projects consisted in 3D-printing individual components that were then vertically stacked. Because of the size constraints of the 3D printers, as well as the limited number of layers that could be supported, the columns were created in sections [29,30]. The additive manufacturing technique used in the production of both columns made it possible to fabricate complex and unique forms that could not be fabricated with conventional molding techniques. The biologically active process, however, introduces new challenges, such as additional sterilization needs for the processing of the materials. In order to overcome these challenges, the designers explored novel manufacturing techniques and tested various material compositions [29]. The manufacturing method used by Blast Studio starts with shredding waste paper coffee cups to generate organic substrates, which are then inoculated with fungal spawn. The generated biomass paste is pushed through an extruder layer by layer to create ten separate modules. These modules are then piled into a column of 2.1 m in height and bio-welded together using more biomass paste [30].

As part of the first study published on the 3D printing of mycelium-based materials on a small scale, Bhardwaj et al. (2020) combined biomass paste with additives after the first growing phase to produce an extrudable mixture [7]. After 3D printing, the printed item was placed in a sterile container for five days for the second stage of growth. Finally, a heater was used to stop the fungus’ growth. The results showed that similar to the results of the molding technique, the majority of mycelial growth occurred in the outer parts [7]. The same group of researchers published a follow-up study in which they investigated the effects of the composition of the mixture, particularly the addition of psyllium husk powder, on the 3D printing quality and the rheological properties of the mixture [20]. In another study, chitosan and shredded bamboo fibers were used for preparing a mycelium mixture. Chitosan is a biopolymer derived from chitin. When dissolved in a moderately acidic liquid medium, it generated a gel that served as a physical stabilizer and facilitated the extrusion of the mycelium paste [11].

In the *MyCera* project [31], a unique form of 3D printable material was developed using a mixture of clay and mycelium-based composites. Two distinct 3D-printed versions of the composite material were created. “Node” elements were developed using mycelium-based composites to deal with tension and shear forces, and “bar” elements were developed with fired clay. Both samples were made up of a combination of sawdust and clay that was inoculated with the fungal spawn.

In “multi-material fabrication” research, researchers investigated a two-phase multi-material manufacturing method to fabricate mycelium-based composites with enhanced porosity and intricacy [32]. The suggested technique bypasses existing 3D printing restrictions by adding a secondary material to act as structural support in order to create the complicated structure for this study. The basic inoculated pulp that makes up the form is extruded, and a fabrication setup is created to deposit the secondary material that supports and forms it.

In another study, several characteristics that determine the 3D printability of mycelium-based composite materials were systematically studied [28]. The viscosity of the mixture, extrusion parameters, and printer hardware settings were the three primary categories of 3D printing parameters that were analyzed. By outlining several substrate compositions, material processing, 3D printing settings, the development of extruders, and sterile printing techniques for living extrudable materials, this work provided clear workflows for the 3D printing of mycelium-based composites.

In the *Tilted Arch* project, the main purpose was to explore the mechanical characteristics of 3D-printed mycelium-based components by 3D-printing a funicular form that only worked under compressive forces [4]. Their findings demonstrated the possibility of creating compression-only forms that can bear loads by 3D printing and bio-welding mycelium-based composite components.

Table 1 provides an overview of the projects mentioned above and reviews all of the variables related to the 3D printing of mycelium-based composites and the six steps of cultivation outlined above in Figure 2.

Similar to molding or other building techniques with mycelium-based materials, it is necessary to first inoculate the primary substrate, let it colonize for a while, and then mix it with other additives to obtain workable extrusion pulp. According to Lim and Thomsen (2021), there are two main criteria to obtain workable extrusion pulp: its ability to sustain fungal growth and development, and its suitability for fabrication, which involves both extrudability and material stability during the 3D printing and growth stages [32].

### 2.5. Contamination and Its Effect on Mycelial Growth

*P*. *ostreatus* (oyster mushroom) was the fungal species chosen to generate grain-based spawn that followed substrate inoculation under axenic conditions (i.e., sterile substrate and aseptic environmental conditions). However, during the second stage of fungal growth, after 3D printing, if the mycelium-based paste is exposed to the environment, it becomes susceptible to microbial contamination. For example, a previous report shows that contamination with the fungus *Trichoderma* sp. (green mold disease) can significantly affect mycelium composites’ growth [34]. It is difficult to distinguish *Trichoderma* growth from mushroom growth during the first days of colonization, since it first develops a thick, pure, white mycelium layer that is similar to a mushroom mycelium. During the reproductive stage, *Trichoderma* produces green structures (spores), which are a clear indicator of this infection, often during the secondary colonization step [35]. Because of the high sporulation of the causative agent, which results in a recognizable infection symptom, the mycelial mat on the covering layer of the mycelium-based composite gradually takes on a green color [36]. Because of the high sporulation of the causative agent, which results in a recognizable infection symptom, the mycelial mat on the covering layer of the mycelium-based composite gradually takes on a green color [37].

The management of this infection is very difficult, since both *P. ostreatus* and *Trichoderma* are fungi. Enzymes produced by *Trichoderma* are antagonistic to many microorganisms, which allows it to inhibit the development of *P. ostreatus* mycelia. Due to the green mold fungi attack, the oyster mushroom mycelium does not grow as expected [38].

## 3. Materials and Methods

The procedure for the experiments that follow comprised three main stages, all of which were completed in succession:

(1) Development of extrudable mycelium-based mixtures.
(2) Monitoring growth and tracking the source of contamination.
(3) Three-dimensionally printing mycelium-based composites.

The cultivation of the material was the first step in the development of an extrudable paste that could flow through the nozzle of the 3D printer. The first step in developing the material was preparing substrate mixtures using cardboard as the primary substrate. The cardboard, which was pulped, was stored in autoclavable bags before being sterilized in an autoclave. The cardboard was sterilized prior to inoculation with *P. ostreatus* spawn. For the first phase of growth, the inoculated bags were kept in an environment regulated with respect to humidity, temperature, and lighting. After 14 days of growth within bags, additives were added to the mixture to make it extrudable for 3D printing purposes. The process of handling the colonized substrate and the addition of additives increases the risk of contamination after the first stage of growth.

### 3.1. Development of Extrudable Mycelium-Based Mixtures with Waste Cardboard as Substrate

The steps for preparing different mycelium-based mixtures are discussed in detail in this section. These steps included processing the waste cardboard to convert it to a primary substrate to cultivate mycelium on, preparing substrates in autoclavable plastic bags, sterilization of the substrates, inoculating the substrates and primary colonization of the mycelium, and preparation of extrudable mixtures.

#### 3.1.1. Collecting and Sorting Waste Cardboard

According to the United States EPA (Environmental Protection Agency), cardboard and paperboard account for the largest percentage of all materials in municipal solid waste. Despite the fact that paper and cardboard are the most recycled materials in 2018, significant quantities of waste cardboard and paper are still ending up in landfills, ranking third among municipal solid waste dump products in the United States [39].

As industrial waste, cardboard is rich in cellulosic and lignocellulosic substances that could be used as nutrients to cultivate and produce mycelium-based materials [40]. Studies have shown that oyster mushrooms (*Pleurotus* spp.) can grow well on cardboard and other lignocellulose-rich substrates [41]. Growing mycelia on cardboard makes it possible to reuse existing material and gives its lifecycle a new purpose before it is discarded [42].

In this research, waste cardboard-based materials were chosen as the substrate materials, since they are a great source of lignin and cellulose for feeding fungi. The cardboard used was collected from recycling bins and studio spaces located throughout Penn State University, Stuckeman School of Architecture and Landscape Architecture. This cardboard would have otherwise been thrown away as waste or recycled into new products if it had not been collected for this research. Before using the cardboard, every label, excess glue, and piece of additional material were eliminated.

Using waste cardboard as a source of nutrients to grow mycelia on makes it possible to reuse a waste product and gives a new purpose to its lifecycle before it is discarded. Using waste cardboard benefits the nature of biodegradable materials, which are capable of decomposition either in the presence of oxygen or in an anaerobic environment, in addition to being more environmentally friendly.

#### 3.1.2. Substrate Preparation

The primary material for this experiment, waste cardboard, was first separated based on sheet size. In order for mycelia to grow over and through the cardboard pieces, the cardboard sheets were first shredded and reduced to sufficiently small sizes ranging from 5 mm to 15 mm. For preparing the cardboard, smaller pieces were processed using an office shredder, while larger pieces were shredded using a Precix 3-axis CNC router with a 4′ × 8′ bed and an engraving drill bit that moved in an iterative pattern. The dust and particles produced during this procedure were also collected with the dust-collecting container connected to the machine.

The waste cardboard pulp was mixed with 10% (*w*/*w*) wheat bran, enhancing mycelial development and boosting cultivation speed, acting as a supplemental nutrient. The water content of the primary material was adjusted to keep the bag’s moisture level at 65%. The substrate mixtures were packed in plastic autoclavable bags of 200 mm × 125 mm × 480 mm. Each bag was tightened using a twist tie and kept in a cold room. Each prepared bag contained 71.5 g of dry-pulled cardboard, 28.5 g of wheat bran (100 g of dry material), and 185 g of water.

#### 3.1.3. Sterilization

Before sterilization, the twist ties were loosened from the bags to ensure that water vapor did not pressurize the bags. The bags were also capped with a paper bag and tape to reduce the interaction between the contents and the environment between sterilization and inoculation.

The bags were then autoclaved for 40 min at 121 °C. Sterilizing in an autoclave using steam under pressure eliminates harmful microorganisms such as molds and pathogens. The bags were then cooled overnight in a cold room at 4 °C. The next day, the paper bags were removed, and the twist ties were tightened to seal the bags.

#### 3.1.4. Mycelium Inoculation and Primary Colonization

The primary substrates were inoculated with *P. ostreatus* spawn. The fungal spawn was purchased in a pre-spawn bag made of supplemented cotton seed hulls and straw from Lambert Spawn (Strain 123 *P. ostreatus*; Coatesville, PA, USA). The formula for adding the spawn was 10% of the dry weight of each bag, which was 10 g (each bag had 100 g of dry material consisting of 71.5 g of pulped cardboard and 28.5 g of wheat bran). The spawn was directly added into the bags, and after the bags were tied, they were thoroughly mixed and shaken by hand. The bags were left to grow in a climate-controlled production room at the Mushroom Research Center at Pennsylvania State University for 14 days. The climate-controlled room provided more precise control over the growth environment with 95% relative humidity, a temperature of 24 ± 1 °C, and no direct sunlight.

#### 3.1.5. Preparation of Extrudable Mixtures

According to Bhardwaj [20], to develop a paste or mixture that is extrudable and has certain characteristics, such as “flowability”, water and a gelling agent need to be added to the colonizing-mycelium mixture in this step. When dissolved in a liquid phase, gelling agents form a mildly cohesive internal structure in a homogeneous mixture. The gelling agent chosen for this study was psyllium husk. Steam-treated Nature’s Promise Whole Psyllium Husk and Now Foods Whole Psyllium Husk Powder were used as gelling agents in different experiments. Before opening, the colonizing-mycelium bags were thoroughly cleaned by spraying them with 70% ethanol solution. Five different mixtures with different colonizing-mycelium (M)-to-distilled water-to-psyllium husk (PH) ratios by weight (M:W:PH) were prepared to test the extrudability of the mixtures, as can be seen in Table 2.

The “flowability” of the mixtures was first tested with a medical syringe and then with a paste extruder (3D Potterbot).

For the medical syringe tests, 50 g of the colonizing mycelium was broken off by hand, with sterilized gloves, at first into smaller parts and then pounded into smaller chunks using mortar and pestle. Then, distilled water and psyllium husk were added in the ratios indicated in Table 2. Syringes were filled by hand with the prepared mixtures. The mixtures were extruded onto plexiglass plates in the form of straight lines, curved lines, and stacked lines on top of each other by pushing the plunger at a steady speed. The plexiglass plate and the syringe were cleaned using 70% ethanol solution prior to the tests.

Moving to a larger scale, 50 g of colonizing mycelium was broken off by hand, with sterilized gloves, into smaller parts and then chopped up using a household kitchen mixer for 30 s at medium speed. Distilled water was added to the mixer, following the ratios indicated in Table 2, and the mixtures were mixed for 20 s at medium speed. The mixer container was manually shaken after every 5 s to ensure consistent contact between the mixer blade and the material throughout the mixing phase. Then, psyllium husk was added to the mixer, following the ratios indicated in Table 2, and was manually blended using a sterilized spoon. The acrylic tubes of the 3D Potterbot paste extruder were filled with the mixtures by hand. A nozzle with a 5 mm diameter was used to test the extrudability of the mixtures on a larger scale. The working table, the tools, and the mixer were carefully cleaned by spraying them 70% with ethanol solution.

Based on the results, *Mixture A*, with a 5:20:2 ratio of colonizing mycelium:distilled water:psyllium husk, was chosen to be used for 3D printing purposes.

### 3.2. Monitoring Growth and Tracking the Source of Contamination

Even when taking careful precautions to minimize the risk of contamination, contaminants can find their way into a mixture. Due to the mixture’s minimal contact with its surroundings during the primary colonization stage, there is little chance that the mycelium mixture gets contaminated during the first growth period. Most contamination is likely to occur in the latter phases of colonization, after the mixtures are extruded. Tracking the source of contamination becomes important at this point to identify ways to mitigate it.

To track possible sources of contamination, samples from different stages of mixture preparation were transferred to Petri dishes, and the growth of *P*. *ostreatus* mycelia and contaminants were visually documented over time.

#### 3.2.1. Isolating the Mixtures from Different Stages of Mixture Preparation to Track the Source of Contamination

The isolation experiment was repeated three times (A, B, and C) using the same primary substrates and adding water and psyllium husk, following the ratio of *Mixture A* shown in Table 2. The mixtures from the four consecutive stages of mixture preparation and extrusion were sampled in four separate, sterile Petri dishes for each set. Table 3 summarizes the contents of the Petri dishes. Round sterile Petri dishes (100 mm × 15 mm; VWR, Ohio, USA) were used. The Petri dishes, after being filled, were enclosed and sealed with Parafilm^®^ (Neenah, WI, USA). Parafilm is a semi-transparent, flexible, thermoplastic film that provides a waterproof barrier for cover. It is gas permeable with low water permeability. It does not affect oxygen or carbon dioxide permeability and does not inhibit mycelial growth.

The first Petri dish included a mixture of 2 g of psyllium husk (PH) and 20 g of distilled water (W), and no colonizing mycelium. The goal was to determine if the source of contamination was psyllium husk or the colonizing mycelium.

The other Petri dishes included colonizing mycelia. Immediately after opening the bags, 100 g of colonizing mycelium (M) was taken out using a sterilized stainless-steel spatula and placed in the mixer. A total of 400 g of distilled water (W) was added and mixed for 30 s. The second Petri dish was filled with 25 g of this mixture, directly taken with a sterile sampler from the mixer. A total of 20 g of psyllium husk (PH) was added to the mixer, and the material was thoroughly mixed. The mixture at this stage was ready for 3D printing. Before filling the acrylic tube of the 3D Potterbot paste extruder for extruding, 27 g of the mixture was sampled in the third Petri dish. The fourth Petri dish was filled with extruded material that went through the 3D Potterbot nozzle.

All of the Petri dishes were kept in a climate-controlled growth room at XXX. They were regularly observed to record changes in growth. Photos of the Petri dishes from the three experiment sets were taken on the 1st, 3rd, 5th, and 7th days of growth in the Petri dishes.

#### 3.2.2. Sterilization of Water and Psyllium Husk

Distilled water (W) and psyllium husk (PH) were separately sterilized in this stage. For the first two sets of Petri dish isolation experiments (A and B), psyllium husk was placed in a large glass container. The container was enclosed with aluminum foil and autoclaved for 30 min at 121 °C. PYREX reusable medium storage bottles were filled to about half of their capacity with distilled water (to prevent water from overflowing during sterilization) and placed in the autoclave chamber. For the third set of Petri dish isolation experiments, a smaller amount of psyllium husk was placed in 50 mL conical plastic centrifuge tubes (VWR, Randor, PA, USA). These disposable conical-bottom sterile tubes have a smooth inner wall for easy filling and unloading and are autoclavable. The cap was left loose to reduce the internal pressure build-up in the tube during sterilization. The tubes were wrapped with aluminum foil to keep the cap sterile until use. All the additives were left to cool down overnight.

### 3.3. Mitigating Contamination

Following the isolation experiments to track the source of contamination, follow-up experiments were performed to explore ways to mitigate contamination. The first experiment was to check if the psyllium husk and water that had been sterilized in the autoclave were contaminant-free or not. The second experiment was to implement several changes in the working protocols and repeat the isolation study outlined in Table 3 in three sets (D, E, and F) without including the last extrusion step, as can be seen in Table 4.

#### 3.3.1. Sterilization Check in PDA

After the sterilization of psyllium husk and water in the autoclave for 30 min at 121 °C, the materials were moved to a laminar airflow workstation and sampled using a steel sterilized sampler into Petri dishes with PDA medium. A Petri dish with a growth medium solidified with agar is called agar plate (PDA). These types of Petri dishes are used to culture microorganisms. The Petri dishes were sealed using parafilm and then stored side up in an incubator at room temperature to keep both culture and agar properly hydrated. The Petri dishes were regularly observed to record changes.

#### 3.3.2. Changes in the Working Protocols

These experiments were performed to explore ways to mitigate contamination. The changes made to the working protocols can be listed as follows:

- A Laminar Airflow Workstation (biosafety hood) was used. All of the materials and tools used were kept under the biosafety hood during the mixing and sampling process. The workstation is an enclosed cabinet designed to prevent sensitive materials from being contaminated. The steel surfaces of the biosafety hood were sanitized by spraying 70% ethanol before working.
- A Waring 700 G blender with a glass container was used for mixing the substrates. The glass container and all of the tools were carefully washed with Alconox detergent, left in the lab to dry, and sprayed with 70% ethanol solution before use.
- A lab alcohol burner was used to produce an open flame for the flame sterilization of a stainless-steel scoop spatula.
- An empty centrifuge tube was used to measure 2 g of sterile psyllium husk for each of the 1D, 1E, and 1F Petri dishes.
- The colonizing-mycelium bag was cut using an aluminum-body cutting knife. The cutting knife was flame-sterilized before use. The colonizing mycelium was taken out of the bag using a sterilized stainless-steel spatula and was directly poured into the sterile glass blender to avoid contact with the hand or any external contamination source.
- The glass bottle containing sterilized water was opened under the biosafety hood, and sterilized water was added to the blender.
- The blender and all of the tools were washed and sterilized between experiment sets (D, E, and F).
- All of the Petri dishes were sealed with parafilm before being moved outside the biosafety hood.
- All of the nine Petri dishes were stored in a box and moved to a climate-controlled room at the Mushroom Research Center. They were regularly observed, and the changes were documented by taking photos on the 1st, 3rd, 5th, and 7th days of growth.

### 3.4. Three-Dimensional Printing of Mycelium-Based Composites with Waste Cardboard

After changes were made to the working protocols and contamination was mitigated, three prototypes of living mycelium-based composites cultivated on waste cardboard were 3D-printed and left to fully grow. Again, mixture samples from different stages of mixture preparation were isolated in Petri dishes.

#### 3.4.1. Three-Dimensional Printing System and Hardware

The 3D printing platform used in these experiments was 3D PotterBot 9XL (Stuart, FL, USA), a 3D clay printer that uses a stationary paste extruder [43]. The acrylic extrusion tube, which can hold up to 2 L of material, is composed of four main parts: nozzle, nozzle adapter, piston, and gearbox adapter. The nozzle is attached to the nozzle adapter, and the adapter is connected to the main tube with screws. Several conical nozzles with diameters of 2 mm, 5 mm, and 9 mm were tested for 3D printing, resulting in the selection of the nozzle with a 5 mm diameter. The acme screw movement is controlled by the acme nut and anti-rotator. The screw is connected to the motor and gearbox with the gearbox adapter. The rotation of the acme screw pushes the piston, resulting in the extrusion of material inside the acrylic tube. The 3D printer is remotely controlled using its internal internet system. Files containing the 3D printing toolpath, gcode, are generated using Cura slicing software, version 5.0.

#### 3.4.2. Computational Workflow

**Figure 3:**
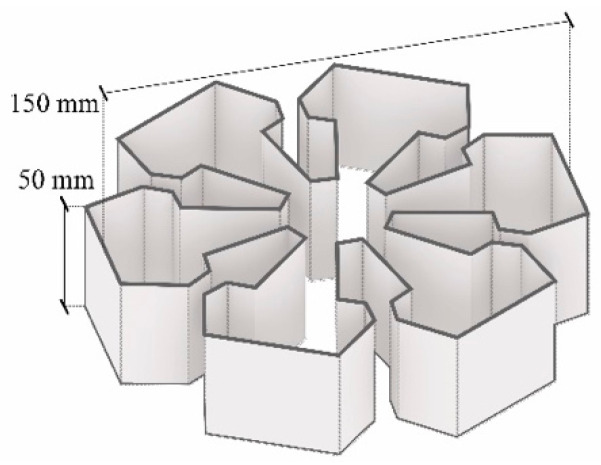
Geometry to 3D print.

The computational workflow included the design and modeling of the geometry and the generation of the *g-code* for the 3D PotterBot system. The geometry was designed with Rhinoceros 7 software as an object with 150 mm diameter and 50 mm height (Figure 3). A simple design approach was chosen in this stage, since the main objective of this paper was to investigate the practicality of 3D printing using mycelium-based materials cultivated on waste paper-based substrates. The 3D PotterBot system used does not allow to stop and start extrusion. Therefore, the geometry was designed to maximize the extruded surfaces within a given volume. The 3D object was then imported into Cura slicing software to generate the g-code. The profile for the specific 3D PotterBot was downloaded, allowing the importing of all of the settings for the 3D printer [43]. The retraction distance of 1000 mm, the layer height of 5 mm, and the print speed of 100 mm/s were used.

#### 3.4.3. Development of a Sterile, Isolated 3D Printing System

**Figure 4:**
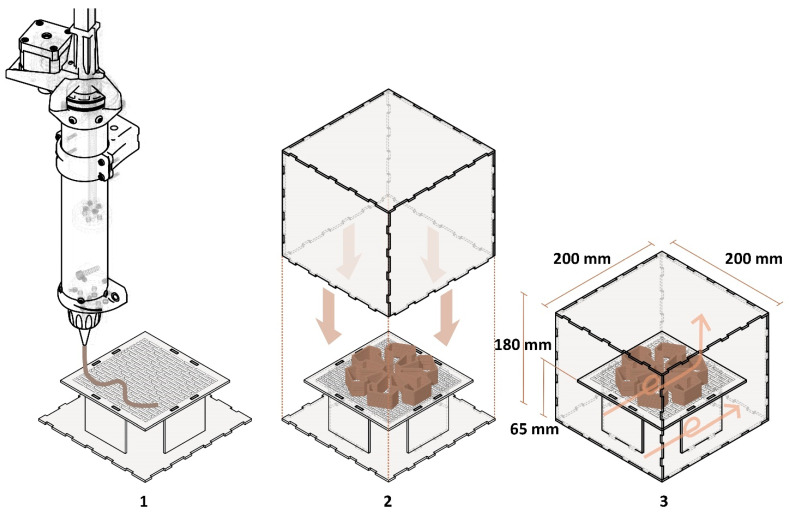
Isolation acrylic box. (**1**) Three-dimensional printing on the printing bed, (**2**) the enclosing of the box, and (**3**) airflow in the box helping mycelial growth.

To have a sterile and isolated environment for the mycelium-based material after 3D printing, an acrylic transparent box was designed (Figure 4). The box was made of 3 mm thick transparent acrylic sheets to be able to observe and document changes in the 3D-printed object inside. The box was 200 mm × 200 mm × 180 mm, with a floating surface inside. The surface inside the box was 180 mm × 180 mm, providing space between the walls for airflow. The surface was perforated by cutting out holes in it, which helped the mycelia also grow on the bottom part of the 3D-printed object by letting air travel through the interior space of the box. The surface was attached to the bottom face of the box using four smaller acrylic pieces. This floating, perforated surface was placed on the printing bed of 3D PotterBot. After 3D printing, the box was enclosed, and all of its edges were sealed with Parafilm.

The isolation box was kept in a climate-controlled growth room at the Mushroom Research Center. It is worth mentioning that all parts of the box were washed using Alconox detergent, a laboratory cleaning solution. The parts were left in the lab to dry, then 70% ethanol solution was sprayed before we started 3D-printing. The parts of 3D PotterBot were also washed with Alconox and sprayed with 70% ethanol solution to make all of the tools and the 3D printing environment sterile.

#### 3.4.4. Drying and Heating 3D-Printed Samples

The 3D-printed samples were taken out of the isolation boxes after 7 days and were left to air-dry with a fan. After 6 h, the samples were placed in an oven at 90 °C for 2 h, which caused the objects to lose their water content and killed the living fungal organisms.

## 4. Results

### 4.1. Extrudability and Growth of the Mixtures

The results are summarized in Table 5. The mixture with the lowest water content, *Mixture E*, could not be extruded using a medical syringe or 3D PotterBot using the 5 mm nozzle. All of the other mixtures could be extruded through the syringe and the 5 mm nozzle. Mixture D had the same ratio of psyllium husk to water (PH:W) as *Mixture C* and *Mixture A* and could be extruded with the medical syringe and the 5 mm nozzle. However, the extrusions were not consistent because of the mixture’s lower colonizing-mycelium-to-water ratio (M:W). The extrusions of *Mixture D* obtained using a syringe completely dried out after 2 days, resulting in no mycelial growth. Samples extruded with 3D PotterBot using *Mixture D* had slight growth. *Mixtures A*, *B*, and *C* were successfully extruded using both the syringe and 3D PotterBot. However, the extrusions of *Mixture B* and *Mixture C* obtained with 3D PotterBot were inconsistent, again, probably because of their lower colonizing-mycelium-to-water ratios (M:W). The extrusions of *Mixture B* and *Mixture C* obtained with the syringe only grew until the third day and then stopped growing, because the extruded samples completely dried out. Comparing the extrudability, consistency, and mycelial growth of all of the mixtures tested, *Mixture A* was chosen for the follow-up experiments in this research. In *Mixture A*, for each 50 g of colonizing mycelium, 200 g of water and 20 g of psyllium husk were added.

### 4.2. Tracking and Mitigating the Source of Contamination

The results of isolating the materials and mixtures to track and mitigate the source of contamination are outlined in three sections: (1) Contamination Source in Mixtures from Different Stages of Mixture Preparation, (2) Effectiveness of Sterilization, and (3) Mitigating Contamination (by changing the working protocols).

#### 4.2.1. Contamination Source in Mixtures from Different Stages of Mixture Preparation

Table 6 shows the photos of Petri dishes from the three replicate sets (A, B, and C) documented on days 1, 3, 5, and 7.

In the first set, set A, the Petri dish with psyllium husk and water, 1A, showed contamination on the 7th day. The very first signs of contamination were visible in this dish starting on the 5th day, and the contaminating agent took over the dish by the 7th day. The second Petri dish, 2A, which contained a mixture of colonizing mycelium and water, was not contaminated by the 7th day, and mycelial growth could be observed. The third Petri dish from the same set, 3A, contained a mixture of colonizing mycelium, water, and psyllium husk. Traces of Trichoderma growth were observable by the 5th day, and the contaminating agent took over the dish by the 7th day. The same was observed in the fourth Petri dish from this set, 4A, which was filled with extruded material after 3D printing.

Replicates B and C yielded similar patterns. All the Petri dishes that contained psyllium husk in all the experiments were contaminated on the 5th or 7th day. No contamination was observed in the Petri dishes that contained mycelia and water (2A, 3A, and 4A).

Overall, the results of these three experiments show the following:

- A total of 100% of the Petri dishes with psyllium husk were contaminated.
- Very early signs of contamination were visible starting on the 5th day.
- The source of contamination was psyllium husk, the tools used for the 3D printing, or the working environment, and not the colonizing mycelium.

#### 4.2.2. Effectiveness of Sterilization

This experiment was useful for determining if the water and psyllium husk used in the extrudable mixtures were contaminated after sterilization or not. The results can be seen in Table 7.

The sterilized psyllium husk mixed with water, after staying in the incubator for 7 days, had no visible signs of contamination. No signs of either bacterial or fungal contamination were observed from the same psyllium husk isolated in the PDA plate either. Similarly, the water isolated in the PDA was not contaminated after 7 days. However, the water from the same bottle sampled after keeping the bottle open under the biosafety hood for about an hour was contaminated with bacteria.

Overall, the results of this experiment demonstrate that autoclaving effectively sterilizes psyllium husk and water. However, the materials can get contaminated from the working environment. Therefore, more strict working protocols need to be implemented to mitigate contamination.

### 4.3. Mitigating Contamination

The results of this experiment show that the changes listed in Section 3.3.2. “Changes in the working protocols“ helped mitigate contamination. The working protocol, in addition to sterilizing the material itself, is a determining factor in the contamination problem. The results of the visual observation of the dishes on day 7 showed no contamination in any of the Petri dishes, as can be seen in Table 8.

#### 4.3.1. Three-Dimensional Printing of Mycelium-Based Mixtures Cultivated with Waste Cardboard

The three 3D-printed samples kept in the isolated acrylic box were fully grown on the 7th day after 3D printing. The growth of the three samples on days 1, 3, 5, and 7 is shown in Table 8. Again, samples from different stages of mixture preparation were isolated in Petri dishes to monitor the growth and any possible contamination, as shown in Table 9.

**Figure 5:**
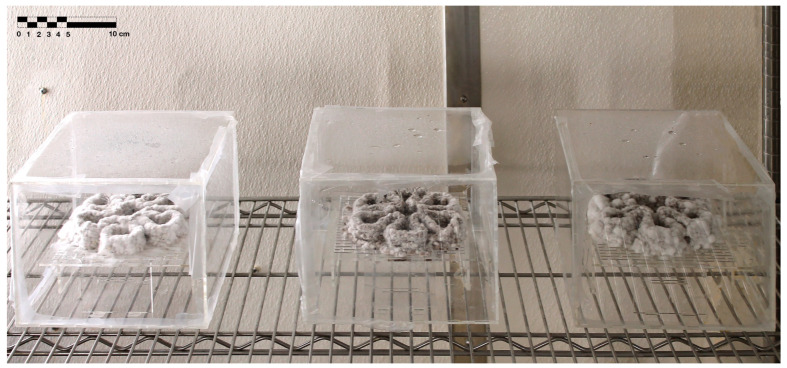
End of secondary colonization of 3D-printed prototypes within acrylic boxes in the environmentally controlled room.

Neither the three 3D-printed prototypes nor the isolated samples in the Petri dishes were contaminated by day 7, and the mycelia had achieved full colonization (Figure 5).

#### 4.3.2. The Sterile, Isolated 3D Printing System

Leaving the 3D-printed object in the isolated plexiglass boxes resulted in isolating the material from the environmental contamination sources on one hand and the growth of mycelia on the bottom part of the 3D-printed object on the other hand.

## 5. Discussion

The results of this study indicate that mycelium-based composites cultivated on waste cardboard demonstrate potential for 3D printing purposes when mixed with psyllium husk and water in a 5:20:2 ratio (colonizing mycelium to water to psyllium husk; M:W:PH). This ratio follows the ratio presented by [7] using the commercial Ecovative (Green Island, NY, USA) mycelium mixture as the basis and validates their research outcomes.

Although it has not been clearly acknowledged and addressed in previous studies, contamination is a major problem in 3D printing mycelium-based composites. However, our study demonstrates that it is possible to mitigate contamination by sterilizing the materials, implementing strict sanitization protocols, and isolating the 3D-printed objects immediately after 3D printing. The results of the isolation experiments indicate that autoclaving as a sanitizing method eliminates the unwanted microorganisms that are harmful to mycelial growth. The sterilization of water and psyllium husk, before mixing with the colonizing mycelium for 3D printing purposes, eliminates the competing organisms. Having said this, the brand of the psyllium husk used may be an important factor in mitigating contamination. Certain brands of psyllium husk, including Nature’s Promise Whole Psyllium Husk, used in this study, are sold “steam-treated, which suggests the potential for the material to contain less contaminants to begin with.

After drying, 3D-printed mycelium-based composites lose about 70% of their water content. This loss causes the deformation and shrinkage of 3D-printed objects. The shrinkage amount can be different if different colonizing-mycelium (M)-to-water (W) ratios in the mixtures or different primary substrates are used.

### 5.1. Limitations of the Study

The 3D printing experiments in this study were small in scale and focused on a simple object. Moving to a larger scale would change some of the variables that may affect the final results. The isolation box technique used for the secondary colonization phase may be hard to employ in larger-scale 3D printing experiments.

In this study, only waste cardboard was used as the primary substrate for mycelium cultivation. A comparative study with other substrates could be performed in the future. In addition, extrudability in this study was assessed visually. Using rheological methods for testing extrudability could result in more accurate results and comparisons.

### 5.2. Contributions

The main contributions of this research can be listed as follows:

- A comparative review of the existing 3D printing efforts for mycelium-based composites to date.
- Recipes for extrudable mycelium mixtures, with waste cardboard as the primary substrate material.
- A method to track the source of possible contamination using isolation in Petri dishes.
- Ways to mitigate contamination using proper sterilization techniques and workflows.

### 5.3. Future Work

In the next steps of this research, we will shift from 3D PotterBot to 3D printing using a six-axis industrial robotic arm, which will provide more freedom of movement and flexibility. The size of the 3D-printed components will be scaled up. In addition, by 3D-printing multiple components that are connected or in relation with each other, the bio-welding of mycelium-based components after 3D printing will be assessed.

Shrinkage can be controlled by predicting the deformation or shape change of objects. By predicting the shrinkage amount, it may be possible to predict the final form of the dried components.

The extrudable mixtures developed in this study can be altered by substituting the primary substrate (waste cardboard) with other organic waste.

Assessing extrudability with scientific methods using rheological studies will help in having more valid results in the future.

## 6. Conclusions

This study explored the potential of mycelium-based composites as sustainable materials for 3D printing purposes. The use of waste cardboard as a substrate for cultivating mycelia and the development of extrudable mixtures and workflows for 3D-printing mycelium-based components were investigated. The study shows the following:

- Waste cardboard is a feasible substrate for cultivating mycelia.
- It is possible to develop extrudable mixtures that can be used for 3D-printing mycelium-based components with waste cardboard as a substrate.
- One of the main challenges to 3D-printing mycelium-based composites is contamination.
- Isolating mixtures from different stages of mixture preparation helps track and mitigate the source of contamination.

The construction industry has long been associated with negative environmental impacts due to the high levels of greenhouse-gas emissions and landfill waste generated during the construction process. As a response, there has been an increasing interest in developing sustainable alternatives to traditional building materials. Mycelium-based composites have emerged as one such alternative with the potential to significantly reduce the environmental impact of the construction industry.

Mycelium-based composites are renewable and biodegradable biomaterials that are obtained with the growth of mycelia (vegetative structures; hyphal networks) on organic substrates such as agricultural waste or even waste cardboard, as demonstrated in this study. Compared with traditional building materials, which are often derived from non-renewable resources and have high levels of embodied energy, mycelium-based composites are a more sustainable alternative that can contribute to a more circular economy.

In comparison to cultivating mycelium-based composites in molds using normal substrates, the following are the significant advancements and distinctive features of 3D-printed mycelium composites:

- The ability to fabricate intricate and complex geometries while reducing formwork waste.
- Contributing to waste reduction and sustainable fabrication practices by using waste cardboard as a primary substrate.

Furthermore, the study addresses the important issue of contamination in 3D-printing mycelium-based composites and proposes solutions such as sterilizing the materials, implementing strict sanitization protocols, and isolating the 3D-printed objects immediately after printing. These measures can help mitigate contamination.

Overall, the findings of this study highlight the potential of mycelium-based composites to be a viable and sustainable alternative to traditional building materials in the construction industry, contributing to a more circular economy and a reduced environmental impact in the construction industry, paving the way for a more sustainable future.

## Figures and Tables

**Table 1:** Existing 3D printing experiments with mycelium-based materials.

	Pulp Faction [29]	Tilted Arch [4]	Mycelium Matters [28]	Mycelium-Bound Composite [11]	MyCera [31]	Multi-Material Fabrication [32]	3D Printing of Biomass-Fungi Composite Material [7]	3D Printing of Biomass-Fungi Composite Material II [20]
	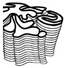	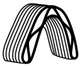	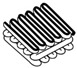	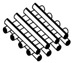	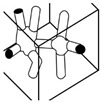	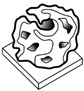	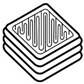	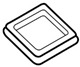
**Sequence** **of steps**	1-2-3-4-5-6	1-2-3-4-5-6	1-2-3-4-5-6	1-2-3-4-5-6	3-4-1-2-5-6	1-2-3-4-5-6	1-2-3-4-5-6	1-2-3-4-5-6
**Step 1: Mycelium Inoculation of Agricultural Feedstock**								
**Mixture**	Fine woodchips + paper pulp + kaolin clay	Shredded paper + wheat bran	Beechwood + paper cellulose	Bamboo fibers (500 μm)	Sawdust (<2 mm) + bleached and unbleached cellulose	Paper pulp + secondary material: sand (0.02–0.05 mm) and gravel (4–8 mm)	Ecovative GIY mixture [33]	Ecovative GIY mixture ([33]
**Ratio**	N/A	65–70 wt% water + 7% wheat bran (dry weight)	64 wt% water	175 g of bamboo fibers + 175 mL of water + 50 g of spawn + sawdust	N/A	N/A	400 g of Ecovative mixture + 700 mL water + 32 g flour	400 g of Ecovative mixture + 700 mL water + 32 g flour
**Fungal strains**	*Byssomerulius corium* + *Gloeophyllum*	*Pleurotus ostreatus* (10% of dry weight content)	*Trametes versicolor + Ganoderma resinaceum*	*Ganoderma lucidum*	*Pleurotus ostreatus*	*Ganoderma Lucidum*	N/A	N/A
**Step 2: Primary Colonization**								
**First incubation period**	7 days	14 days	14 days	1 to 4 weeks	N/A	N/A	3–5 days	3–5 days
**Step 3: Mixing**								
**Gelling agent**	Kaolin Clay	Guar gum	Xanthan gum (3 wt%)	Chitosan	Clay	Xanthan gum	Psyllium husk powder	Psyllium husk powder
**Ratio**	N/A	3:50 Guar gum:water	10–80 wt% fiber-water /5–85 wt% fiber-water	60:40 and 70:30 fiber ratios + 3 wt% chitosan solution at pH~6	7:1 clay: sawdust + 35 wt% water	11% spawn, 56% paper pulp, 1% xanthan gum, and 32% water (by weight)	100 g of primary material + 400 g of water + psyllium husk (20 g)	100 g of primary material + 400 g of water + psyllium husk (0, 10 g, 20 g, or 30 g)
**Method**	N/A	N/A	20 min at 400 rpm	Pounded with pestle and mortar	Mixing machine	N/A	Commercial mixer for 30 s	Commercial mixer for 15 s
**Step 4: 3D Printing**								
**Printer**	Vormvrij Lutum v4	Custom extruder	KUKA KR 15/2 6-axis industrial robot	Manually with serological syringes	Delta WASP 40100	N/A	Delta WASP 2040	Delta WASP 2040
**Nozzle diameter**	3.5 mm	9 mm	N/A	6 mm	4 mm	N/A	4 mm	N/A
**Layer height**	1.5 mm	N/A	3 mm	N/A	N/A	N/A	N/A	N/A
**Print speed**	N/A	N/A	N/A	N/A	N/A	N/A	15 mm/s + air pressure of 3.5 bar	30 mm/s
**Step 5: Secondary Colonization**								
**Second** **incubation period**	N/A	5 weeks + 7 days of biowelding	7 days at 26 °C	20 days	2 weeks at 24 °C	14 days	3–5 days	N/A
**Step 6: Drying/Heating**								
**Drying period**	N/A	N/A	N/A	Overnight at 40 °C	6 h at 600 °C + 2.5 h at 960 °C	N/A	4 h at 95 °C	N/A
**Shrinkage**	40%	N/A	N/A	3–6%	N/A	N/A	N/A	N/A

**Table 2:** Mixtures’ composition.

Colonized Mycelium: Distilled Water: Psyllium Husk M:W:PH (by Weight)	
Mixture A	5:20:2
Mixture B	10:20:1
Mixture C	5:10:1
Mixture D	10:10:1
Mixture E	10:5:1

**Table 3:** Contents of the Petri dishes from the isolation experiment.

Petri Dishes #1A, #1B, and #1C	Petri Dishes #2A, #2B, and #2C	Petri Dishes #3A, #3B, and #3C	Petri Dishes #4A, #4B, and #4C
Psyllium husk + water PH (2 g) + W (20 g)	Colonizing mycelium + water M (5 g) + W (20 g)	Colonizing mycelium + water + psyllium husk M (5 g) + W (20 g) + PH (2 g)	Extruded Colonizing mycelium + water + psyllium husk M (5 g) + W (20 g) + PH (2 g)

**Table 4:** Contents of the Petri dishes in the follow up experiment.

Petri Dishes #1D, #1E, and #1F	Petri Dishes #2D, #2E, and #2F	Petri Dishes #3D, #3E, and #3F
Psyllium husk + water PH (2 g) + W (20 g)	Colonizing mycelium + water M (5 g) + W (20 g)	Colonizing mycelium + water + psyllium husk M (5 g) + W (20 g) + PH (2 g)

**Table 5:** Results of extruding different mixtures.

Extrusion Method	Mixture	Extrudability	Consistency	Mycelial Growth
Medical syringe	Mixture A	Extruded	Consistent	Fully grown
	Mixture B	Extruded	Consistent	Slight growth
	Mixture C	Extruded	Consistent	Slight growth
	Mixture D	Extruded	Inconsistent	No growth
	Mixture E	Not extruded	-	-
3D PotterBot (5 mm nozzle)	Mixture A	Extruded	Consistent	Fully grown
	Mixture B	Extruded	Inconsistent	Fully grown
	Mixture C	Extruded	Inconsistent	Fully grown
	Mixture D	Extruded	Inconsistent	Slight growth
	Mixture E	Not extruded	-	-

**Table 6:** Visual inspection of the mixtures isolated in Petri dishes.

Experiment		1st Day	3rd Day	5th Day	7th Day
A	**Petri dish #1A** Psyllium husk + water W (20 g) + PH (2 g)	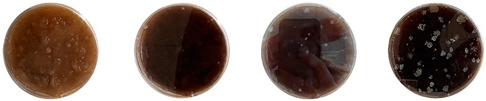			
	**Petri dish #2A** Colonizing mycelium + water M (5 g) + W (20 g)	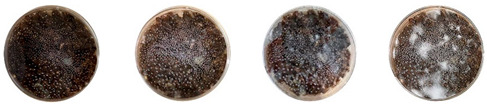			
	**Petri dish #3A** Colonizing mycelium + water + psyllium husk M (5 g) + W (20 g) + PH (2 g)	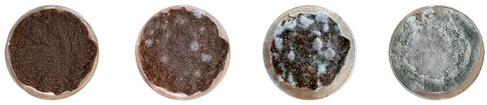			
	**Petri dish #4A** Extruded Colonizing mycelium + water + psyllium husk M (5 g) + W (20 g) + PH (2 g)	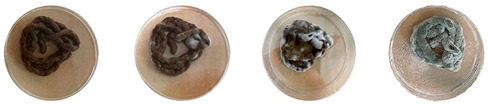			
B	**Petri dish #1B** Psyllium husk + water W (20 g) + PH (2 g)	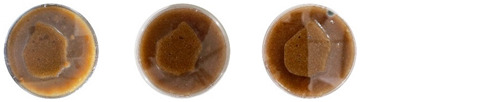			
	**Petri dish #2B** Colonizing mycelium + water M (5 g) + W (20 g)	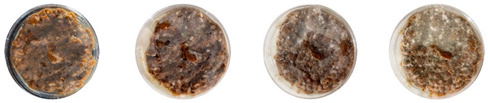			
	**Petri dish #3B** Colonizing mycelium + water + psyllium husk M (5 g) + W (20 g) + PH (2 g)	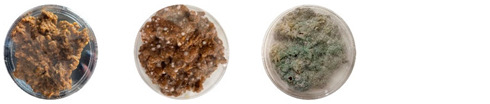			
	**Petri dish #4B** Extruded Colonizing mycelium + water + psyllium husk M (5 g) + W (20 g) + PH (2 g)	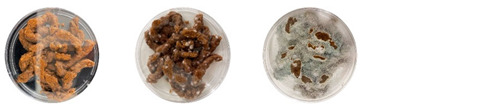			
C	**Petri dish #1C** Psyllium husk + water W (20 g) + PH (2 g)	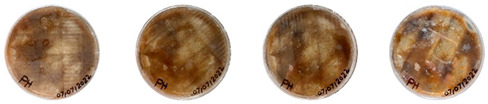			
	**Petri dish #3C** Colonizing mycelium + water + psyllium husk M (5 g) + W (20 g) + PH (2 g)	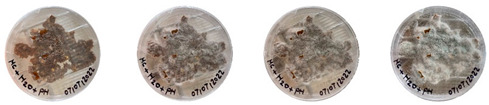			
	**Petri dish #4C** Extruded Colonizing mycelium + water + psyllium husk M (5 g) + W (20 g) + PH (2 g)	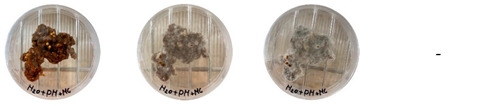			

**Table 7:** Visual inspection of the mixtures isolated in PDA plates.

Psyllium Husk + Water W (20 g) + PH (2 g)	Psyllium Husk on PDA PH (2 g)	Water on PDA W (20 g)	Water on PDA (after Working) W (20 g)
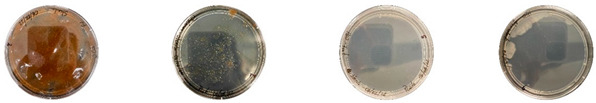			

**Table 8:** Visual inspection of the mixtures isolated in Petri dishes after changing working protocols.

Experiment		1st Day	3rd Day	5th Day	7th Day
D	**Petri dish #1D** Psyllium husk + water W (20 g) + PH (2 g)	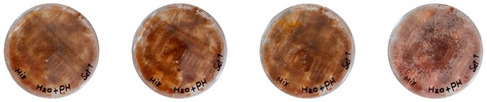			
	**Petri dish #2D** Colonizing mycelium + water M (5 g) + W (20 g)	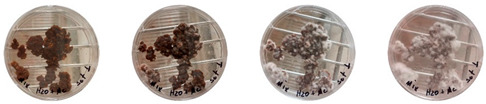			
	**Petri dish #3D** Colonizing mycelium + water + psyllium husk M (5 g) + W (20 g) + PH (2 g)	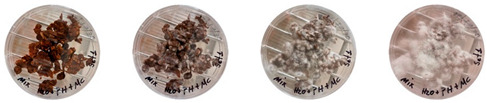			
E	**Petri dish #1E** Psyllium husk + water W (20 g) + PH (2 g)	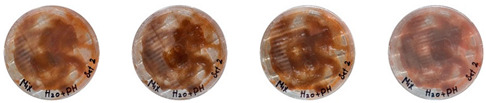			
	**Petri dish #2E** Colonizing mycelium + water M (5 g) + W (20 g)	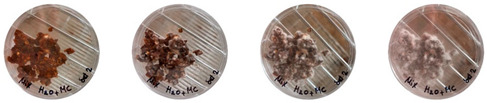			
	**Petri dish #3D** Colonizing mycelium + water + psyllium husk M (5 g) + W (20 g) + PH (2 g)	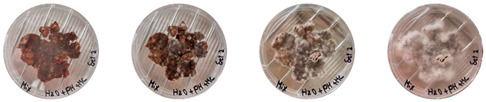			
F	**Petri dish #1E** Psyllium husk + water W (20 g) + PH (2 g)	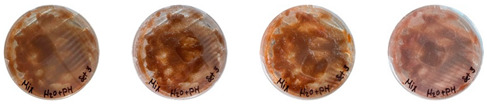			
	**Petri dish #2E** Colonizing mycelium + water M (5 g) + W (20 g)	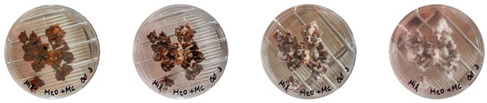			
	**Petri dish #3F** Colonizing mycelium + water + psyllium husk M (5 g) + W (20 g) + PH (2 g)	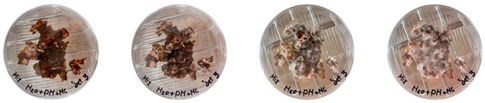			

**Table 9:** Visual inspection of the 3D-printed samples and mixtures isolated in Petri dishes.

Experiment		1st Day	3rd Day	5th Day	7th Day
3D-Printed prototypes	**3D-Printed prototype #1**	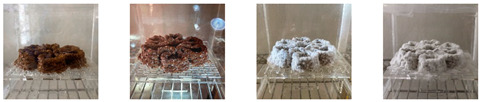			
	**3D-Printed prototype #2**	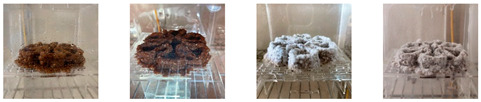			
	**3D-Printed prototype #3**	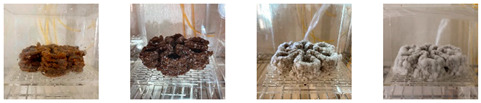			
G	**Petri dish #1G** Psyllium husk + water W (20 g) + PH (2 g)	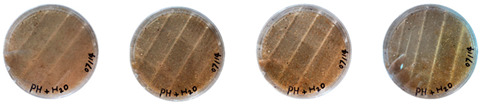			
	**Petri dish #2G** Colonizing mycelium + water M (5 g) + W (20 g)	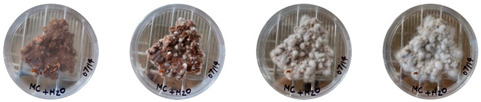			
	**Petri dish #3G** Colonizing mycelium + water + psyllium husk M (5 g) + W (20 g) + PH (2 g)	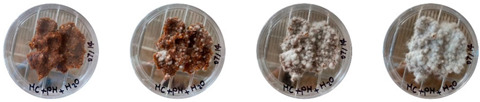			
	**Petri dish #4C** Extruded Colonizing mycelium + water + psyllium husk M (5 g) + W (20 g) + PH (2 g)	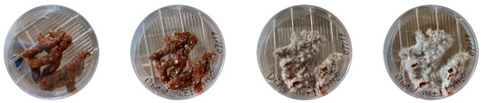			

## Data Availability

No new data were created or analyzed in this study. Data sharing is not applicable to this article.

## References

[1] Girometta C., Picco A.M., Baiguera R.M., Dondi D., Babbini S., Cartabia M., Pellegrini M., Savino E. (2019). Physico-Mechanical and Thermodynamic Properties of Mycelium-Based Biocomposites: A Review. Sustainability.

[2] United Nations Environment Programme (2022). 2020 Global Status Report for Buildings and Construction: Towards a Zero-Emission, Efficient and Resilient Buildings and Construction Sector. https://globalabc.org/sites/default/files/inline-files/2020%20Buildings%20GSR_FULL%20REPORT.pdf.

[3] Almpani-Lekka D., Pfeiffer S., Schmidts C., Seo S. (2021). A review on architecture with fungal biomaterials: The desired and the feasible. Fungal Biol. Biotechnol..

[4] Modanloo B., Ghazvinian A., Matini M., Andaroodi E. (2021). Tilted Arch; Implementation of Additive Manufacturing and Bio-Welding of Mycelium-Based Composites. Biomimetics.

[5] Appels F., Camere S., Montalti M., Karana E., Jansen K.M.B., Dijksterhuis J., Krijgsheld P., Wosten H. (2018). Fabrication factors influencing mechanical, moisture- and water-related properties of mycelium-based composites. Mater. Des..

[6] Gantenbein S., Colucci E., Käch J., Trachsel E., Coulter F., Rühs P.A., Masania K., Studart A. (2023). Three-dimensional Printing of Mycelium Hydrogels into Living Complex Materials. Nat. Mater..

[7] Bhardwaj A., Vasselli J., Lucht M., Pei Z., Shaw B., Grasley Z., Wei X., Zou N. (2020). 3D Printing of Biomass-Fungi Composite Material: A Preliminary Study. Manuf. Lett..

[8] Yang L., Park D., Qin Z. (2021). Material Function of Mycelium-Based Bio-Composite: A Review. Front. Mater..

[9] Ngo T.D., Kashani A., Imbalzano G., Nguyen K.T.Q., Hui D. (2018). Additive manufacturing (3D printing): A review of materials, methods, applications and challenges. Compos. Part B Eng..

[10] Block P., Mele T., Liew A., DeJong M., Escobedo D., Ochsendorf J. (2018). Structural design, fabrication and construction of the Armadillo vault. Struct. Eng..

[11] Soh E., Chew Z.Y., Saeidi N., Javadian A., Hebel D., Ferrand H.L. (2020). Development of an extrudable paste to build mycelium-bound composites. Mater. Des..

[12] Islam M.R., Tudryn G., Bucinell R., Schadler L., Picu R.C. (2017). Morphology and mechanics of fungal mycelium. Sci. Rep..

[13] Jones M.P., Huynh T., Dekiwadia C., Daver F., John S. (2017). Mycelium Composites: A Review of Engineering Characteristics and Growth Kinetics. J. Bionanosci..

[14] Haneef M., Ceseracciu L., Canale C., Bayer I.S., Heredia-Guerrero J.A., Athanassiou A. (2017). Advanced Materials from Fungal Mycelium: Fabrication and Tuning of Physical Properties. Sci. Rep..

[15] Jambaro A., Neri K., Alvarez L. (2014). Utilization of Selected Urban Wastes as Substrate Solutions in the Growth and Yield Performance of *Pleurotus sajor-caju* Fr. (Singer) (Gray *Oyster mushroom*). PUP J. Sci. Technol..

[16] Ghazvinian A., Farrokhsiar P., Rocha Vieira F., Pecchia J., Gursoy B. (2019). Mycelium-Based Bio-Composites for Architecture: Assessing the Effects of Cultivation Factors on Compressive Strength. Mater. Res. Innov..

[17] Attias N., Danai O., Abitbol T., Tarazi E., Ezov N., Pereman I., Grobman Y.J. (2020). Mycelium bio-composites in industrial design and architecture: Comparative review and experimental analysis. J. Clean. Prod..

[18] Grünewald J., Parlevliet P., Altstädt V. (2017). Manufacturing of thermoplastic composite sandwich structures: A review of literature. J. Thermoplast. Compos. Mater..

[19] Yang Z., Zhang F., Still B., White M., Amstislavski P. (2017). Physical and Mechanical Properties of Fungal Mycelium-Based Biofoam. J. Mater. Civ. Eng..

[20] Bhardwaj A., Rahman A.M., Wei X., Pei Z.J., Truong D., Lucht M., Zou N. (2021). 3D Printing of Biomass–Fungi Composite Material: Effects of Mixture Composition on Print Quality. J. Manuf. Mater. Process..

[21] Ghazvinian A. (2021). A Sustainable Alternative to Architectural Materials: Mycelium-based Bio-Composites. Proceedings of the Divergence in Architectural Research.

[22] Grimm A., Eilertsen L., Chen F., Huang R., Atterhem L., Xiong S. (2021). Cultivation of *Pleurotus ostreatus* Mushroom on Substrates Made of Cellulose Fibre Rejects: Product Quality and Spent Substrate Fuel Properties. Waste Biomass Valorization.

[23] Abhijith R., Ashok A., Rejeesh C.R. (2018). Sustainable packaging applications from mycelium to substitute polystyrene: A review. Mater. Today Proc..

[24] Jones M.P., Huynh T., John S. (2018). Inherent species characteristic influence and growth performance assessment for mycelium composite applications. Adv. Mater. Lett..

[25] Long W.-J., Tao J.-L., Lin C., Gu Y., Mei L., Duan H.-B., Xing F. (2019). Rheology and buildability of sustainable cement-based composites containing micro-crystalline cellulose for 3D-printing. J. Clean. Prod..

[26] Elsacker E. (2021). Mycelium Matters—An Interdisciplinary Exploration of the Fabrication and Properties of Mycelium-Based Materials. Ph.D. Thesis.

[27] Ghazvinian A., Khalilbeigi Khameneh A., Mottaghi E., Gursoy B. A Computational Framework for the Design and Fabrication of Spatial Structures with Mycelium-based Composites. Proceedings of the IASS Annual Symposia.

[28] Elsacker E., Peeters E., De Laet L. (2022). Large-scale robotic extrusion-based additive manufacturing with living mycelium materials. Sustain. Futur..

[29] Goidea A., Andreen D., Floudas D. (2020). Pulp Faction: 3D Printed Material Assemblies through Microbial Biotransformation.

[30] Hahn J. (2022). Blast Studio 3D Prints Column from Mycelium to Make “Architecture That Could Feed People”. https://www.dezeen.com/2022/01/18/blast-studio-tree-column-mycelium-design/.

[31] Jauk J., Vašatko H., Gosch L., Christian I., Klaus A., Stavric M. Digital Fabrication of Growth Combining digital manufacturing of clay with natural growth of mycelium. Proceedings of the 26th International Conference of the Association for Computer-Aided.

[32] Lim A.C.S., Thomsen M.R. Multi-material Fabrication for Biodegradable Structures: Enabling the printing of porous mycelium composite structures. Proceedings of the eCAADe 2021: Towards a New, Configurable Architecture.

[33] Grow It Yourself^TM^ Material Grow.bio. https://grow.bio/products/grow-it-yourself-material.

[34] Innocenti G., Montanari M., Righini H., Roberti R. (2019). Trichoderma species associated with green mould disease of *Pleurotus ostreatus* and their sensitivity to prochloraz. Plant Pathol..

[35] Park M.S., Bae K.S., Yu S.H. (2006). Two New Species of Trichoderma Associated with Green Mold of Oyster Mushroom Cultivation in Korea. Mycobiology.

[36] Danesh Y.R., Goltapeh E.M., Rohani H. (2000). Identification of Trichoderma species causing green mould in button mushroom farms, distribution and their relative abundance. Sci. Cultiv. Edible Fungi.

[37] Cao Z.-J., Qin W.-T., Zhao J., Liu Y., Wang S.-X., Zheng S.-Y. (2022). Three New Trichoderma Species in Harzianum Clade Associated with the Contaminated Substrates of Edible Fungi. J. Fungi.

[38] Pakeerthan K. Eco-Friendly Management Common Lab Contaminant *Trichoderma* spp. in *Oyster mushroom* Production Using Agrobased Industry’s by-Products. Proceedings of the International Conference on Climate Change.

[39] (2021). National Overview: Facts and Figures on Materials, Wastes and Recycling. https://www.epa.gov/facts-and-figures-about-materials-waste-and-recycling/national-overview-facts-and-figures-materials.

[40] Owaid M., Abed A., Nassar B. (2015). Recycling cardboard wastes to produce blue oyster mushroom *Pleurotus ostreatus* in Iraq. Emir. J. Food Agric..

[41] Bellettini M.B., Fiorda F.A., Maieves H.A., Teixeira G.L., Ávila S., Hornung P.S., Júnior A.M., Ribani R.H. (2019). Factors affecting mushroom *Pleurotus* spp.. Saudi J. Biol. Sci..

[42] Nguyen M., Ranamukhaarachchi S. (2019). Study on the mycelium growth and primordial formation of king oyster mushroom (*Pleurotus eryngii*) on cardboard and spent coffee ground. Res. Crops.

[43] 3D Potter Frequently Asked Questions (FAQ) about Clay 3D Ceramic Printers and 3D Potterbot—3D Potter Real Clay 3D Ceramic Printers. https://3dpotter.com/faq-v789.

